# Case of Delirium, in Which Large Doses of Opium Were Successfully Employed

**Published:** 1830-02

**Authors:** Joseph Ashley Gaitskell


DELIRIUM.

*Case of Delirium, in which large Doses of Opium were successfully employed.* By Joseph Ashley Gaitskell, M.D.

A tradesman of this city, widower, with six children, fifty-two years of age, tall, boney, very robust frame, and subject to occasional attacks of gout in his feet, acquired unexpectedly a sum of money, which enabled him to retire from business, and buy a country house, horse and gig, and, in short, to set up for a gentleman, thinking the sum inexhaustible. After expending a large sum in improving his purchase, it suddenly entered his mind to ask himself what would be the end of all this? The reflection disturbed him, and he became delirious. Although his property was still considerable for a man of his station in society, the delusion made him apprehensive of seeing himself and his children reduced to poverty; and his fears were aggravated by the thought of being brought to that unhappy state by his own folly. His feelings were so distressing that he threatened to destroy himself, and his manner and conversation were such as justly to alarm his family. During three or four weeks he had no sleep night or day; he was continually pacing his room, and incessantly lamenting his folly; conscious of his delirium, but incapable of controlling his thoughts or actions. In this state he went alone to London, where he remained a fortnight, restricting his diet to a few herrings. On his return, the distress of his mind was aggravated to such an extreme degree, that in his agony he wrote to a friend, entreating him to come and save him from self-destruction, and accused himself, in indefinite terms, of having committed some most dreadful deed. His friend came instantly to his rescue, and found him as above described. Having looked into his affairs, and finding there was little ground for this extreme despondency, and that argument was useless in restoring him to reason, his friend called on me to visit him, with a view to placing him in confinement, considering him as a lunatic.

I saw him in the evening of November 11th, ult. I found his mind as described: he was sweating profusely; pulse seventy-two, soft, and rather weaker than natural; tongue extremely furred; bowels relaxed. He recognised me immediately, but positively refused assistance, saying "it was useless, his case was hopeless; medicine could do him no good; whoever sent me must pay me, for he could not." However, by the persuasion of his friend, he consented to take what I thought proper. I prescribed Camphor gr. iv.; Ext. Hyosciami gr. viij.; Potass. Nitrat. ℈ss. quartis horis sumend. He took four doses in the course of seven hours, and passed the night better, dozing a little.

In the morning he was certainly better; but having finished his medicine, it had been discontinued some hours, and, at the time I saw him, he was in nearly the same state as on the previous evening. I desired the medicine to be continued every two hours, till he had taken eight doses more. At times he was much better, but the amendment was transient, and he still had but little sleep.

Next morning he was rather more rational. He complained of weight on the top of his head; said he had suffered from it more than a fortnight; and it was that pain or weight which took away his reason, and made him not know or say what he did. I ordered him Jalap gr. viij.; Sulphate of Magnesia ʒij. in Infus. Rosæ and Aq. Piment. It operated twice. He now became much worse, and alarmed his family more than ever; a convincing proof that all depletion was hurtful.

In the evening I ordered eight pills, with three grains of purified opium in each, one to be taken every two hours till sleep supervened. By mistake he took two pills at a dose, making twenty-grains of purified opium in ten hours, four and with the happiest effect, producing sound sleep, from which he awoke in the morning as perfectly in his senses as ever he was in his life. The weight or pain at the top of his head was entirely removed. It may be supposed that the opium, being in a solid state, did not wholly dissolve in his stomach; but this was not the case, for, as it was very hard, the druggist was obliged to reduce it to powder, and make it into pills with conserve. Some vomiting occurred two hours after the last dose, which the patient attributed to taking a double dose of the hyosciamus and camphor, intermediate with the pills, unobserved by his attendants.

I now gradually withdrew the opium, by ordering one three-grain pill every two hours for that day; the following days I lengthened the periods, and reduced the quantities so as ultimately to dispense with it altogether; his recovery being complete.

The bowels becoming costive, I ordered jalap and aloes, each two and a half grains, every four hours: he took ten pills, which operated twice. Aloes is a very useful conjunct with opium, when required, as the one does not impede the operation of the other. In this case I ordered the aperient and the opium to be alternated every two hours: each was thus taken separately. Diet was ordered to be light, but nourishing, and, by his own choice, consisted chiefly of beef-tea and poultry.

*Observations*. Had this man been bled largely and much purged, his recovery would probably have been very protracted, even had he escaped with life. In the great majority of cases of this sort on record, bleeding and purging had been freely employed, anterior to the administration of opium. It must, therefore, be doubtful how far the previous treatment contributed to the recovery of the patient, by preparing them for opium. This case proves, as much as one instance can, that such previous treatment is at least not always necessary.

The quantity of opium taken in a short time is very remarkable, and leads to the question, what was the physical state of this patient who took, with benefit to his health, a quantity of opium, sufficient to have destroyed himself, and one or two other men, under ordinary circumstances? I think we must look for the explanation in the state of the nervous system, and that the disproportionate consumption of the nervous energy, or that peculiar something supplied by the nervous system, was the real and only difference; so that, in the event of death, no lesion of any structure would have been found in the brain. And it is as easy to comprehend the death of an individual from an inordinate exhaustion of nervous energy, in whatever that may consist, as from excessive loss of blood from outward wound. In the latter case, no one would expect to find disease of the heart, nor, in the former, ought we to expect change of structure in the brain. This nervous exhaustion is easily explained by the long privation of sleep, and intense thought consequent to the reflection on his extreme folly: it was at the same time aggravated by a considerable diminution of food, with increased bodily exercise. His usual habit was to live well, but he was not addicted to excesses of any kind.

The moral treatment is, I think, of much importance, and should be of the most soothing description, with as little constraint as possible. In this case I desired the patient might be allowed to walk about the house at his pleasure, but be carefully watched, and sharp weapons removed from his reach. The necessity of these cautions was apparent, by his getting up one night to the garret window, which he opened wide, and possibly would have thrown himself out, had he not been followed by his attendant.

No words can express too strongly the impropriety of consigning such cases to a madhouse. In general such patients are docile, little disposed to injure others, and, with watchful care, may be cured without having recourse to a measure which is hurtful to the feelings of the sufferer to the end of his days.

When, by the power of medicines, reason has in some degree resumed its empire, the consolations of religion may be advantageously employed to aid and confirm her dominion, especially when, as in this case, attendance on public worship had been for years constantly neglected.

I cannot conclude without offering my humble acknowledgment to Dr. Abercrombie, for his invaluable work on the Diseases of the Brain, &c. and to the editors of the London Medical and Physical Journal, for having supplied us with the additions in the second edition. In works of this merit, I think it would be very acceptable to the possessors of the first edition, if the improvements of succeeding editions were made accessible to the purchasers of the first.

Justice requires we should give Dr. Sutton the credit of having first made the profession acquainted with the utility of opium in these cases; although he candidly acknowledges that he acquired the practice from others.

